# The burden of low back pain and its association with socio-demographic variables in the Middle East and North Africa region, 1990–2019

**DOI:** 10.1186/s12891-023-06178-3

**Published:** 2023-01-23

**Authors:** Saeid Safiri, Seyed Aria Nejadghaderi, Maryam Noori, Mark J. M. Sullman, Gary S. Collins, Jay S. Kaufman, Catherine L. Hill, Ali-Asghar Kolahi

**Affiliations:** 1 Social Determinants of Health Research Center, Tabriz University of Medical Sciences, Tabriz, Iran; 2 Research Center for Integrative Medicine in Aging, Aging Research Institute, Tabriz University of Medical Sciences, Tabriz, Iran; 3 School of Medicine, Shahid Beheshti University of Medical Sciences, Tehran, Iran; 4 Systematic Review and Meta-analysis Expert Group (SRMEG), Universal Scientific Education and Research Network (USERN), Tehran, Iran; 5 Student Research Committee, School of Medicine, Iran University of Medical Sciences, Tehran, Iran; 6 Department of Life and Health Sciences, University of Nicosia, Nicosia, Cyprus; 7 Department of Social Sciences, University of Nicosia, Nicosia, Cyprus; 8 Centre for Statistics in Medicine, NDORMS, Botnar Research Centre, University of Oxford, Oxford, UK; 9 NIHR Oxford Biomedical Research Centre, Oxford University Hospitals NHS Foundation Trust, Oxford, UK; 10 Department of Epidemiology, Biostatistics and Occupational Health, Faculty of Medicine, McGill University, Montreal, QC, Canada; 11 Rheumatology Unit, The Queen Elizabeth Hospital, Woodville, South Australia Australia; 12 Discipline of Medicine, University of Adelaide, Adelaide, Australia; 13 Social Determinants of Health Research Center, Shahid Beheshti University of Medical Sciences, Tehran, Iran

**Keywords:** Low back pain, Prevalence, Incidence, Epidemiology, Middle East and North Africa

## Abstract

**Background:**

Low back pain (LBP) is the most common musculoskeletal disorder globally. Providing region- and national-specific information on the burden of low back pain is critical for local healthcare policy makers. The present study aimed to report, compare, and contextualize the prevalence, incidence and years lived with disability (YLDs) of low back pain in the Middle East and North Africa (MENA) region by age, sex and sociodemographic index (SDI), from 1990 to 2019.

**Methods:**

Publicly available data were obtained from the Global Burden of Disease (GBD) study 2019. The burden of LBP was reported for the 21 countries located in the MENA region, from 1990 to 2019. All estimates were reported as counts and age-standardised rates per 100,000 population, together with their corresponding 95% uncertainty intervals (UIs).

**Results:**

In 2019, the age-standardised point prevalence and incidence rate per 100,000 in MENA were 7668.2 (95% UI 6798.0 to 8363.3) and 3215.9 (95%CI 2838.8 to 3638.3), which were 5.8% (4.3 to 7.4) and 4.4% (3.4 to 5.5) lower than in 1990, respectively. Furthermore, the regional age-standardised YLD rate in 2019 was 862.0 (605.5 to 1153.3) per 100,000, which was 6.0% (4.2 to 7.7) lower than in 1990. In 2019, Turkey [953.6 (671.3 to 1283.5)] and Lebanon [727.2 (511.5 to 966.0)] had the highest and lowest age-standardised YLD rates, respectively. There was no country in the MENA region that showed increases in the age-standardised prevalence, incidence or YLD rates of LBP over the measurement period. Furthermore, in 2019 the number of prevalent cases were highest in the 35–39 age group, with males having a higher number of cases in all age groups. In addition, the age-standardised YLD rates for males in the MENA region were higher than the global estimates in almost all age groups, in both 1990 and 2019. Furthermore, the burden of LBP was not associated with the level of socio-economic development during the measurement period.

**Conclusion:**

The burden attributable to LBP in the MENA region decreased slightly from 1990 to 2019. Furthermore, the burden among males was higher than the global average. Consequently, more integrated healthcare interventions are needed to more effectively alleviate the burden of low back pain in this region.

**Supplementary Information:**

The online version contains supplementary material available at 10.1186/s12891-023-06178-3.

## Introduction

Low back pain (LBP) is one of the leading causes of health loss and disability around the world [1, 2]. Furthermore, LBP imposes an enormous economic burden on not only the affected individuals and their families, but also on the communities and governments, due to reduced productivity and the number of work days lost [3]. A study of the United States Claims Database (2006–2008) found that the total direct costs due to chronic LBP with neuropathic pain was US$96 million over a 12-month follow-up period [4]. According to the Global Burden of Disease (GBD) 2017 study, LBP is the most prevalent and greatest cause of disability among all musculoskeletal disorders [5] and in 2019 caused the ninth largest disease burden, globally [6].

As a result of population growth and aging, the physical and economic costs attributed to LBP are projected to rise in the future, highlighting the need for rapid healthcare actions to be undertaken, in particular in regions with poorly equipped infrastructures [7]. LBP is an important global health concern and deserves much more epidemiological research at the local level to inform policy makers with current detailed data and trends over time. In 2019, globally LBP was found to be more common among females and its highest point prevalence was among those aged 80–84 years old [8]. This same research found that globally there was a weak positive association between the burden of LBP and socioeconomic development [8].

A previous GBD 2019 project reported the burden of LBP at the global, regional and national levels [8]. However, stratified levels have not been previously reported for the MENA countries by sex, age group, or according to the level of sociodemographic development [8]. Furthermore, the two previous publications using the GBD 2017 [9] and GBD 2010 [10] data to report the worldwide burden of LBP, with no details reported for individual countries and territories. Consequently, there is a need to investigate the LBP attributable burden at the national level and for the provision of more detailed information. Therefore, the present study used GBD 2019 data to report the point prevalence, annual incidence, and years lived with disability (YLD) that were attributable to LBP for the 21 countries located in the MENA region, from 1990 to 2019, by sex, age, and socio-demographic index (SDI).

## Methods

### Overview

GBD 2019 collected data from 1990 to 2019 on the burden of 369 diseases and injuries and 87 risk factors in 204 countries and territories, seven super-regions (i.e., High income; Latin America & Caribbean; Sub-Saharan Africa; North Africa & Middle East; South East Asia, East Asia & Oceania; South Asia; and Central Europe, Eastern Europe & Central Asia) and 21 regions [11]. As one of the most commonly studied diseases, the burden of LBP has been estimated for all regions of the world. The present systematic analysis reports the burden of LBP, from 1990 to 2019, for those countries within the MENA region. The MENA region consists of: Afghanistan, Algeria, Bahrain, Egypt, Iran, Iraq, Jordan, Kuwait, Lebanon, Libya, Morocco, Oman, Palestine, Qatar, Saudi Arabia, Sudan, the Syrian Arab Republic, Tunisia, Turkey, the United Arab Emirates and Yemen. A detailed description of the methodology used to estimate the burden of LBP in GBD 2019 can be found elsewhere [11] and the raw data can be obtained using the following websites: https://vizhub.healthdata.org/gbd-compare/ and http://ghdx.healthdata.org/gbd-results-tool. The study is based on the Guidelines for Accurate and Transparent Health Estimates Reporting (GATHER) statement (Table S1).

### Case definition and data sources

The “low back” is the area on the posterior aspect of the body, from the lower margin of the twelfth ribs to the lower gluteal folds [11] and LBP is low back pain (with or without pain referred into one or both lower limbs) that lasts for at least 1 day [11]. The International Classification of Diseases (ICD) codes for version 9 (724) and version 10 (M54.3, M54.4 and M54.5) for LBP were used [11].

The most recent systematic review of the literature was conducted by IHME from October 2016–October 2017 and searched the following electronic databases: PubMed, Ovid Medline, EMBase, and CINAHL. The individual search terms used were: “back pain,” “lumbar pain,” “back ache,” “backache,” and “lumbago” and these were also combined with each of the following: “prevalence,” “incidence,” “cross-sectional,” and “epidemiology.” Articles were excluded if they did not report original data (e.g. reviews, commentaries and letters), were not population-based, did not use representative samples or had small sample sizes (< 150). The reason for excluding articles with low sample sizes was to avoid biased estimates that may result from a small sample. Additional details are available in the GBD capstone paper [11]. Further information was obtained from studies that were encountered during the data review and from GBD’s survey database (Global Health Data Exchange - GHDx), which includes both World Health surveys and national health surveys. Furthermore, claims data from the USA, by state, were included for 2000, 2010–2012, and 2014–2016. A detailed description of the data sources and modeling process can be found here: https://ghdx.healthdata.org/gbd-2019/data-input-sources.

### Data processing and disease model

Where possible, the prevalence estimates were split according to sex and age. However, if prevalence was reported for broad age groups by sex (e.g., separately reporting 15–60 year old males and 15–60 year old females), or according to specific age groups without splitting males and females (e.g., 15–30-years and 31–65 year old, for both sexes), the age-specific estimates were separated by sex using the sex ratio reported in the study and the bounds of uncertainty. In cases that did not provide a within-study sex ratio, they were split using a sex ratio derived from a meta-analysis of LBP studies using MR-BRT (a meta-regression tool). The overall female to male ratio was 1.18 (1.18 to 1.18). Finally, where studies reported estimates across age groups of 25 years or greater, DisMod-MR 2.1 was used to divide the estimates into five-year age groups using the prevalence age pattern estimated in GDB 2017.

DisMod-MR 2.1 was also used to model the prevalence and incidence of LBP. In the DisMod model, excess mortality was set to 0, and there was no incidence of LBP before 5 years of age. The modeling strategy from GBD 2017 and the same country-level covariates were used in GBD 2019 [11].

### Years lived with disability

The GBD disability weight (DW) survey was used to provide lay descriptions of the sequelae and to highlight the most important functional consequences and symptoms of LBP [11]. Table S2 presents the severity levels, lay descriptions and DWs of LBP. The disability adjusted life years (DALY) is a metric that is commonly used to quantify the burden of a disease [15], and is produced by combining the years of life lost due to premature mortality and the YLDs [11]. As there was no evidence of LBP-related deaths, the YLD and DALY estimates were the same [11]. The LBP-related YLDs were calculated by multiplying the severity-specific prevalence estimates with their corresponding DWs. All estimates included 95% uncertainty intervals (UIs), which were produced by sampling 1000 draws at each computational step and by combining uncertainty from several sources (e.g., input data, corrections of measurement error, and estimates of residual non-sampling error). The UIs were comprised of the 25th and 975th values of the ordered draws.

### Compilation of results

Smoothing splines models were used to examine the relationship between the LBP-related YLDs and the SDIs for the 21 countries in the MENA region [12]. The SDI (range 0 = less developed to 1 = most developed) consists of the: 1) gross domestic product per capita, smoothed over the last decade; 2) average years of schooling in the population aged 15 and over; and 3) the total fertility rate under 25 years of age. The age groups included in the study were: 5–9, 10–14, 15–19, 20–24, 25–29, 30–34, 35–39, 40–44, 45–49, 50–54, 55–59, 60–64, 65–69, 70–74, 75–79, 80–84, 85–89, 90–94, and 95 plus. R software (V. 3.5.2) was used to conduct all statistical analyses.

## Results

### The Middle East and North Africa region

In 2019, there were 43.2 million (95% UI: 37.8 to 48.8) prevalent cases of LBP in the MENA region, with an age-standardised point prevalence of 7668.2 (6798.0 to 8363.3) per 100,000 population, which is 5.8% lower than in 1990 (4.3 to 7.4) (Table 1 and Table S3). LBP accounted for 18.3 million (16.1 to 20.9) incident cases in 2019, with an age-standardised rate of 3215.9 (2838.8 to 3638.3) per 100,000 population, which is 4.4% lower than in 1990 (3.4 to 5.5) (Table 1 and Table S4). Also in 2019, the number of YLDs due to LBP was 4.9 million (3.4 to 6.5), with an age-standardised rate of 862.0 (605.5 to 1153.3) YLDs per 100,000 population, which has decreased by 6.0% since 1990 (4.2 to 7.7) (Table 1 and Table S5).

**Table 1:** Prevalent cases, incident cases and YLDs due to low back pain in 2019 and percentage change of age-standardised rates during 1990–2019. (Generated from data available from http://ghdx.healthdata.org/gbd-results-tool)

	Prevalence (95% UI)			Incidence (95% UI)			YLDs (95% UI)		
	Counts (2019)	ASRs (2019)	Pcs in ASRs 1990–2019	Counts (2019)	ASRs (2019)	Pcs in ASRs 1990–2019	Counts (2019)	ASRs (2019)	Pcs in ASRs 1990–2019
**North Africa and Middle East**	**43,239,039** **(37,773,370, 48,819,120)**	**7668.2** **(6798, 8636.3)**	**− 5.8** **(− 7.4, − 4.3)**	**18,336,870** **(16,080,397, 20,926,523)**	**3215.9** **(2838.8, 3638.3)**	**− 4.4** **(− 5.5, − 3.4)**	**4,899,300** **(3,404,485, 6,533,347)**	**862** **(605.5, 1153.3)**	**−6** **(− 7.7, − 4.2)**
**Afghanistan**	**1,757,626** **(1,520,204, 2,005,763)**	**7307** **(6427, 8281.8)**	**0.9** **(− 2.8, 4.9)**	**781,631** **(677,351, 895,750)**	**3108.2** **(2743.7, 3521.5)**	**0.6** **(− 2.4, 3.5)**	**196,023** **(136,064, 264,247)**	**803.6** **(568, 1075.4)**	**1** **(− 3.6, 5.7)**
**Algeria**	**2,938,809** **(2,560,184, 3,336,911)**	**7237.5** **(6353.5, 8175.9)**	**− 1.9** **(− 5.8, 1.9)**	**1,257,427** **(1,097,529, 1,445,308)**	**3081.2** **(2713.3, 3498)**	**−1.8** **(− 4.7, 1.1)**	**333,813** **(230,155, 447,252)**	**816.5** **(567.8, 1094)**	**− 2.1** **(− 6.3, 2.3)**
**Bahrain**	**120,196** **(102,199, 139,069)**	**7213.6** **(6350.1, 8149)**	**−1** **(− 4.7, 3.1)**	**51,265** **(43,818, 59,721)**	**3082.6** **(2707.7, 3504)**	**−1** **(− 4.3, 2.1)**	**13,742** **(9477, 18,769)**	**808.9** **(568.9, 1083.6)**	**−1.2** **(− 5.5, 3.2)**
**Egypt**	**6,364,141** **(5,533,169, 7,233,281)**	**7461.2** **(6540.7, 8414.6)**	**2.2** **(− 1.6, 6.5)**	**2,738,368** **(2,402,636, 3,135,984)**	**3155.7** **(2789.9, 3568.5)**	**1.3** **(− 1.6, 4.3)**	**724,668** **(504,744, 974,424)**	**842.9** **(590.7, 1130.1)**	**2.2** **(− 2.2, 6.9)**
**Iran (Islamic Republic of)**	**7,415,044** **(6,524,554, 8,393,689)**	**8486.5** **(7533.8, 9528.3)**	**− 9.5** **(− 10.6, − 8.3)**	**3,050,709** **(2,686,650, 3,487,116)**	**3493** **(3092.5, 3949.5)**	**− 8.4** **(− 9.2, − 7.5)**	**837,779** **(583,888, 1,128,834)**	**951.9** **(668.9, 1275.2)**	**− 9.5** **(− 10.7, − 8.2)**
**Iraq**	**2,476,373** **(2,157,322, 2,802,292)**	**7172.3** **(6322.7, 8116.5)**	**− 3.1** **(− 6.5, 0.6)**	**1,079,909** **(949,882, 1,240,336)**	**3058.3** **(2709.3, 3476.3)**	**− 2.6** **(− 5.4, 0)**	**279,601** **(193,899, 377,243)**	**802.3** **(560.9, 1078.3)**	**− 2.6** **(− 6.6, 1.3)**
**Jordan**	**717,146** **(622,952, 816,397)**	**7253.9** **(6391.6, 8222.1)**	**− 1.1** **(− 4.7, 2.5)**	**312,024** **(271,793, 358,356)**	**3094.6** **(2725.9, 3511.3)**	**− 1.1** **(− 4.1, 1.9)**	**81,779** **(56,682, 108,985)**	**819.8** **(570.5, 1091.2)**	**− 1** **(− 4.9, 3.2)**
**Kuwait**	**346,361** **(297,418, 398,409)**	**7412** **(6519.9, 8339.5)**	**0.3** **(− 3.5, 4.4)**	**147,167** **(126,465, 171,814)**	**3140.3** **(2778.6, 3552.8)**	**− 0.2** **(− 3.3, 2.5)**	**39,639** **(26,978, 53,817)**	**835.7** **(589.3, 1121)**	**− 0.1** **(− 4.3, 4.3)**
**Lebanon**	**346,477** **(304,628, 392,389)**	**6493.5** **(5721.5, 7349.7)**	**− 0.8** **(− 4.2, 2.8)**	**150,708** **(132,419, 171,751)**	**2830.3** **(2484.6, 3214.3)**	**− 1** **(− 4, 1.8)**	**38,867** **(27,267, 52,121)**	**727.2** **(511.5, 966)**	**− 1** **(− 4.7, 3.1)**
**Libya**	**490,208** **(423,628, 561,130)**	**7103.7** **(6233.9, 8024.1)**	**− 2.4** **(− 6.2, 1.3)**	**210,985** **(183,586, 243,608)**	**3042** **(2684.6, 3459)**	**− 2.2** **(− 5, 1.1)**	**55,476** **(38,326, 75,270)**	**796.3** **(558.9, 1069.2)**	**−3.1** **(− 7.1, 1.1)**
**Morocco**	**2,770,031** **(2,428,118, 3,142,593)**	**7717.5** **(6818.5, 8677.7)**	**2.4** **(−1.6, 6.6)**	**1,165,371** **(1,027,188, 1,327,666)**	**3230.5** **(2860.1, 3658.1)**	**1.3** **(− 1.5, 4.3)**	**313,322** **(220,856, 423,475)**	**868** **(610.1, 1167)**	**2.2** **(− 2.2, 6.9)**
**Oman**	**307,584** **(259,823, 357,611)**	**7209.6** **(6333.4, 8186.6)**	**− 0.9** **(− 4.7, 3.4)**	**133,636** **(113,171, 158,197)**	**3087.2** **(2716.5, 3502.3)**	**− 0.9** **(− 4, 2.4)**	**35,426** **(24,025, 48,333)**	**812.7** **(568.2, 1092.1)**	**− 0.9** **(− 5.3, 3.9)**
**Palestine**	**265,697** **(232,238, 301,139)**	**7102.3** **(6257.6, 7999.9)**	**− 3.4** **(− 7.1, 0.3)**	**117,259** **(102,576, 134,388)**	**3042.9** **(2687.5, 3462.5)**	**−2.7** **(− 5.6, 0.7)**	**29,959** **(20,907, 39,885)**	**793.3** **(560.7, 1063.7)**	**− 3.7** **(− 7.6, 0.6)**
**Qatar**	**222,755** **(188,144, 258,441)**	**7483.1** **(6566.5, 8458.3)**	**0.1** **(− 3.8, 4.7)**	**95,630** **(81,013, 114,716)**	**3184.1** **(2795.4, 3622.6)**	**− 0.4** **(− 3.7, 2.9)**	**25,695** **(17,541, 35,014)**	**839.5** **(592.4, 1132.8)**	**− 0.2** **(− 4.8, 5.1)**
**Saudi Arabia**	**2,553,569 (2,178,915, 2,947,793)**	**7170.1** **(6283.2, 8113.1)**	**0.3** **(− 3.3, 4.1)**	**1,104,496** **(943,692, 1,291,967)**	**3071.7** **(2708.8, 3479.2)**	**− 0.2** **(− 3.3, 2.9)**	**291,699** **(199,992, 397,000)**	**803** **(568.3, 1071.7)**	**0** **(− 4, 4.4)**
**Sudan**	**2,093,653** **(1,817,650, 2,382,925)**	**7006.6** **(6190.2, 7926.1)**	**− 1.3** **(− 4.7, 2.3)**	**927,368** **(803,298, 1,067,815)**	**3010.1** **(2654, 3429.2)**	**− 1.2** **(− 4, 1.4)**	**237,414** **(164,627, 316,341)**	**788.3** **(553.5, 1045.1)**	**− 1.3** **(− 5.2, 2.6)**
**Syrian Arab Republic**	**1,007,021** **(871,605, 1,147,942)**	**7160.2** **(6255, 8108.7)**	**− 4.2** **(− 8, − 0.2)**	**433,623** **(378,477, 495,509)**	**3056.3** **(2686.2, 3463.2)**	**− 3.6** **(− 6.3, − 0.8)**	**113,375** **(79,653, 152,271)**	**802.3** **(559.6, 1070.6)**	**−5** **(− 9.1, − 0.7)**
**Tunisia**	**897,946** **(783,942, 1,024,027)**	**7022.1** **(6160.7, 7987.7)**	**0.4** **(− 3.4, 4.6)**	**382,940** **(337,333, 438,013)**	**3009.4** **(2660.6, 3420.7)**	**−0.1** **(− 3.2, 3.5)**	**101,525** **(71,422, 135,332)**	**791.5** **(553.5, 1052.4)**	**0.1** **(− 4.2, 5)**
**Turkey**	**7,730,835** **(6,788,734, 8,691,678)**	**8453.5** **(7457.1, 9478.7)**	**− 14.2** **(− 19.5, − 9.2)**	**3,140,050** **(2,767,927, 3,566,643)**	**3452.6** **(3052.3, 3899.1)**	**−9.5** **(− 13.4, − 5.8)**	**874,588** **(608,052, 1,178,266)**	**953.6** **(671.3, 1283.5)**	**−14.4** **(− 19.9, − 8.9)**
**United Arab Emirates**	**770,334** **(641,479, 919,311)**	**7101.6** **(6223.6, 8047.2)**	**− 0.5** **(− 4.9, 4.4)**	**330,457** **(271,005, 401,767)**	**3053** **(2672, 3475.2)**	**− 0.8** **(− 4.2, 2.9)**	**89,026** **(60,322, 122,762)**	**798.9** **(559.5, 1067.8)**	**−0.7** **(− 5.5, 4.4)**
**Yemen**	**1,603,302** **(1,388,518, 1,831,446)**	**7208.7** **(6336, 8120.3)**	**−5.4** **(− 9.1, − 1.8)**	**707,218** **(614,181, 811,161)**	**3072.8** **(2702.7, 3471.5)**	**−4.4** **(− 7.3, − 1.2)**	**180,905** **(124,718, 242,346)**	**807.2** **(563.7, 1075.2)**	**−5.4** **(− 9.3, − 1.4)**

### National level

**Fig. 1:**
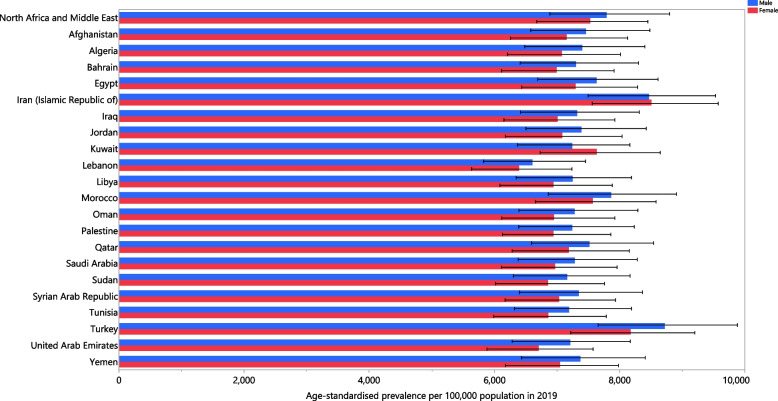
Age-standardised point prevalence of low back pain (per 100,000 population) in the Middle East and North Africa region in 2019, by sex and country. (Generated from data available from http://ghdx.healthdata.org/gbd-results-tool)

In 2019, the national age-standardised point prevalence of LBP ranged from 6493.5 to 8486.5 cases per 100,000 population in the MENA countries. Iran [8486.5 (7533.8 to 9528.3), Turkey [8453.5 (7457.1 to 9478.7)] and Morocco [7717.5 (6818.5 to 8677.7)] had the three highest age-standardised point prevalence rates in 2019. In contrast, Lebanon [6493.5 (5721.5 to 7349.7)], Sudan [7006.6 (6190.2 to 7926.1)] and Tunisia [7022.1 (6160.7 to 7987.7)] had the lowest rates (Table S3). The national age-standardised point prevalences of LBP in 2019 are reported for males and females in Fig. 1.

The national age-standardised incidence rate of LBP in 2019 varied from 2830.3 to 3493.0 cases per 100,000 population. The highest rates were observed in Iran [3493.0 (3092.5 to 3949.5)], Turkey [3452.6 (95% UI: 3052.3 to 3899.1)] and Morocco [3230.5 (2860.1 to 3658.1)], while the lowest rates were found in Lebanon [2830.3 (2484.6 to 3214.3)], Tunisia [3009.4 (2660.6 to 3420.7)] and Sudan [3010.1 (2654.0 to 3429.2)] (Table S4). The national age-standardised incidence rates of LBP in 2019, for males and females, are presented in Fig. S1.

In 2019, the national age-standardised YLD rate of LBP ranged from 727.2 to 953.6 cases per 100,000 population. The highest rates were observed in Turkey [953.6 (671.3 to 1283.5)], Iran [951.9 (668.9 to 1275.2)] and Morocco [868.0 (610.1 to 1167.0)]. Conversely, the lowest rates were seen in Lebanon [727.2 (511.5 to 966.0)], Sudan [788.3 (553.5 to 1045.1)] and Tunisia [791.5 (553.5 to 1052.4)] (Table S5). The national age-standardised YLD rates of LBP in 2019, for males and females, are reported in Fig. S2.

**Fig. 2:**
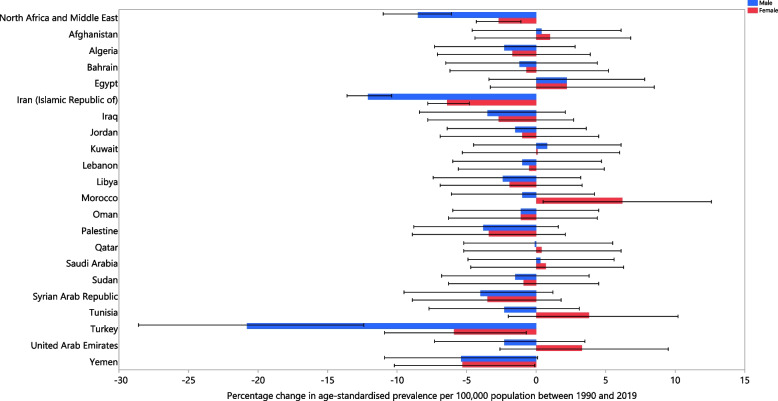
The percentage change in the age-standardised point prevalence of low back pain in the Middle East and North Africa region from 1990 to 2019, by sex and country. (Generated from data available from http://ghdx.healthdata.org/gbd-results-tool)

There were no significant increases in the age-standardised prevalence, incidence or YLD rate of LBP in any of the MENA countries over the period 1990 to 2019. Turkey (Prevalence: [− 14.2 (− 19.5 to − 9.2)]; Incidence: [− 9.5 (− 13.4 to − 5.8)]; YLD: [− 14.4 (− 19.9 to − 8.9)]), Iran (Prevalence: [− 9.5 (− 10.6 to − 8.3)]; Incidence: [− 8.4 (− 9.2 to − 7.5)]; YLD: [− 9.5 (− 10.7 to − 8.2)]) and Yemen (Prevalence: [− 5.4 (− 9.1 to − 1.8)]; Incidence: [− 4.4 (− 7.3 to − 1.2)]; YLD: [− 5.4 (− 9.3 to − 1.4)]) had the largest decreases within the measurement period (Table S3-S5). The percentage change in the national age-standardised point prevalence, from 1990 to 2019, is reported in Fig. 2. In addition, the incidence and YLDs for males and females are reported in Fig. S3 and Fig. S4, respectively.

### Age and sex patterns

**Fig. 3:**
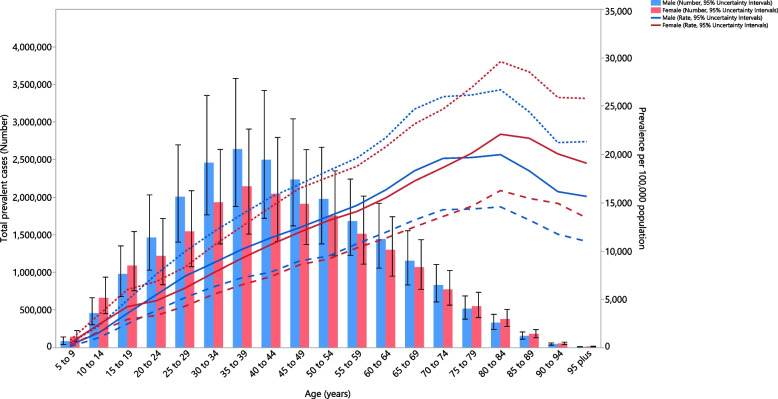
Numbers of prevalent cases and prevalence of low back pain per 100,000 population in the Middle East and North Africa region, by age and sex in 2019; Dotted and dashed lines indicate 95% upper and lower uncertainty intervals for the point prevalence per 100,000 population, respectively. The solid lines represent the point estimation for the point-prevalence per 100,000 population (Generated from data available from http://ghdx.healthdata.org/gbd-results-tool)

In 2019, the point prevalence of LBP in the MENA region was highest in the 80–84 age group, for both males and females, while the number of prevalent cases were highest in the 35–39 age group. Furthermore, the point prevalence and number of prevalent cases of LBP in the MENA region were higher in males of all ages (Fig. 3). In 2019, the corresponding incidence rate was highest in the 75–79 and 80–84 age groups, for males and females, respectively. The number of incident cases of LBP reached its highest in the 35–39 age group for both males and females (Fig. S5). In addition, the YLD rate was highest in the 70–74 and 80–84 age groups, for males and females, respectively. The number of YLDs also reached its highest in the 35–39 age group for both males and females (Fig. S6). Again, the incidence and YLDs, in terms of number and rate, were higher in males of all ages.

**Fig. 4:**
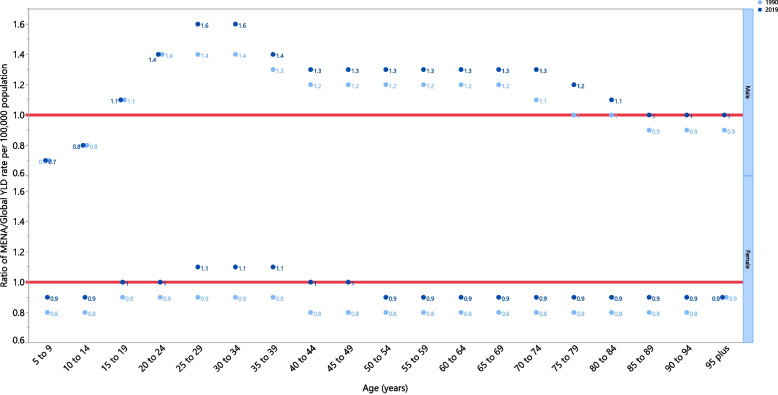
Ratio of the Middle East and North Africa region to the global low back pain YLD rate by age and sex, 1990 and 2019. YLD = years lived with disability. (Generated from data available from http://ghdx.healthdata.org/gbd-results-tool)

The rate ratio, comparing the age-standardised YLD rates in MENA with the global rates for each age group and sex, in 1990 and 2019, showed that there were substantial variations from the global rates. Specifically, in the MENA region males aged 15–84 years old had a higher burden of LBP in both 1990 and 2019. In contrast, females had an equal or lower burden of LBP, in both 1990 and 2019, in almost all age groups (except 25–39 years old in 2019) (Fig. 4).

### Association with the socio-demographic index (SDI)

**Fig. 5:**
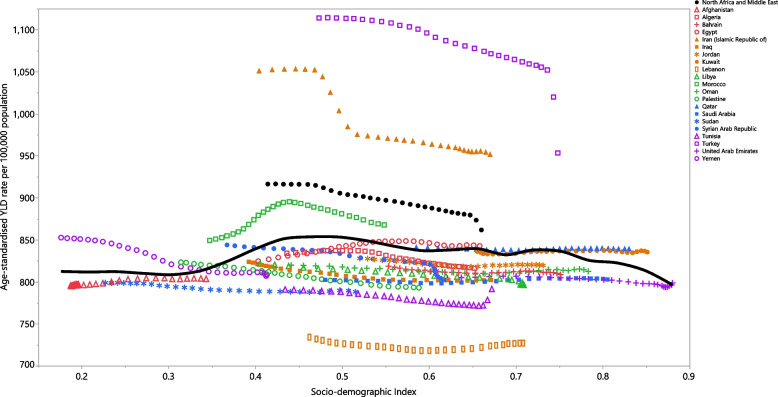
Age-standardised YLD rates of low back pain for 21 countries and territories, by SDI 1990–2019; Expected values based on the Socio-demographic Index and disease rates in all locations are shown as the black line. Each point shows the observed age-standardised YLD rate for each country during 1990–2019. YLD = years lived with disability. SDI = Socio-demographic Index (Generated from data available from http://ghdx.healthdata.org/gbd-results-tool)

The burden of LBP was not associated with socio-economic development over the measurement period. Turkey, Iran and Morocco had much higher than expected burdens from 1990 to 2019, while Lebanon, Tunisia, Sudan, Iraq, Oman, Saudi Arabia, Bahrain, Jordan, Libya, Algeria, Oman and Afghanistan had lower than expected burdens (Fig. 5).

## Discussion

The present study reported the burden of LBP in the MENA region from 1990 to 2019 and found decreases in the age-standardised point prevalence (5.8%), annual incidence (4.4%) and YLD (6.0%) rates. There were no large variations between countries, each estimate was within the other’s UI, meaning that these are not statistically different. Therefore, a reasonable conclusion is that the point prevalence, incidence and YLD were all approximately homogeneous within the MENA countries. Moreover, the age-standardised rate of LBP increased slightly with advancing age and reached a peak around the eighth and ninth decades of life, while no substantial association was found with SDI. Interestingly, the YLD rate among adult males was higher than at the global level, whereas the burden was lower for females in almost all age groups in both 1990 and 2019.

Using GBD 2019 data, a recent article on the global burden of LBP reported that the global age-standardised point prevalence, incidence and YLD rates of LBP in 2019 were 6972.5, 2748.59 and 780.2, respectively [8]. The corresponding figures for the MENA region were 7668.2, 3215.9 and 862.0 per 100,000 population (age-standardised prevalence, incidence and YLD rates, respectively), demonstrating a higher burden than the global average. Furthermore, the percentage changes in the age-standardised rates in the prevalence (− 5.8% vs. -0.16%), incidence (− 4.4 vs. -0.13%) and YLD (− 6.0% vs. -0.16%) were also higher than the global average [8]. The greater burden of LBP in the MENA region could be explained by a higher prevalence of risk factors, such as occupational exposures, smoking, high body mass index (BMI), and lower physical activity, than the global average [13, 14]. In 2016, it was reported that the ergonomic risk factors for LBP were responsible for 21.5% of the occupational attributable DALYs in the MENA region, which was 1.2% greater than the global value for the same year [15]. Interestingly, during the period of study (i.e., 1990–2019), the global reduction in the prevalence of smoking was much higher than in MENA (− 27.5% vs. -11.2% for males and − 37.7% vs. -2.9% for females) [16]. Furthermore, the MENA region had one of the highest increases in smoking among children aged 5–19 years between 1975 and 2016 [17]. Furthermore, in 2014 the prevalence of excess body weight (BMI > 25) was found to be higher in MENA, for both males and females, than the global average [18]. In addition, the countries located in Central Asia and MENA had the highest non-communicable disease burden that was attributable to physical inactivity, accounting for 32.8% of all attributable risks in these countries [19].

At the national level, Iran and Turkey had the highest age-standardised prevalence, incidence and YLD rates. A cohort study conducted on 163,770 Iranians from the general population found that the lifetime prevalence of LBP was 25.2% and that being overweight [odds ratio (OR): 1.13 (95% confidence interval (CI): 1.07–1.19)] or obese [OR: 1.21 (1.16–1.27)], a former smoker [OR: 1.25 (1.16–1.36)] or a current smoker [OR: 1.28 (1.17–1.39)] and having low physical activity [OR: 1.07 (1.01–1.14)] were all significantly associated with the occurrence of LBP [14]. Iran had a higher prevalence of low physical activity than the Eastern Mediterranean Region (EMR) average (55% vs. 31%) [20]. Moreover, Iran was one of the countries that had an increase in the age-standardised prevalence of smoking among females (8.3%) and males (2.3%), despite a decrease in the age-standardised prevalence rate in the MENA region [16]. In addition, regarding occupational LBP, a systematic review reported that neglecting basic ergonomic principles in workplaces and a lack of effective interventions were major risk factors among Iranian workers [21]. Therefore, the higher burden of LBP in Iran could be due to the higher prevalence of risk factors in this country. Furthermore, a cross-sectional study, conducted on 7897 participants in the Trabzon province in Turkey, showed that smoking, low educational attainment and underlying chronic diseases were risk factors for LBP [22]. In light of the above, interventions in these countries should focus on reducing smoking and ergonomic stressors, as well as increasing physical activity in order to control and reduce the burden of LBP. Moreover, educational programs, such as holding ergonomic training courses and advising individuals to remain active, as well as non-pharmacological therapies, like cognitive behavioral therapy, spinal manipulation, massage, acupuncture, yoga and interdisciplinary rehabilitation could be effective in the prevention and management of LBP [7]. Furthermore, while Turkey and Iran were the MENA countries with the highest age-standardised YLD rates of LBP in 2019, they also experienced the largest decrease from 1990 to 2019. These decreases may be as a result of the implementation of programs to prevent LBP or to manage LBP properly. Therefore, it is suggested that these programs are continues, in addition to the abovementioned measures.

Previous research has reported the global burden of LBP to be higher among women than men in almost all age groups, although the differences in the prevalence, incidence and YLDs were not significant [8]. Although we also found that the burden of LBP did not differ discernibly by sex, the prevalence, incidence and YLDs were higher in males across all age groups. In accordance with our findings, the GBD 2013 study also reported that males had higher DALYs for LBP in the EMR (911.5 vs. 827.3 per 100,000 population) [23]. However, in 2017 the age-standardised point prevalence of LBP in MENA was higher in females than among males (10.8% vs. 9.1%) [9]. One reason for the higher number of cases of LBP in MENA, than worldwide, could be the higher number of individuals of working age in the MENA region, since LBP is 2.5 times more prevalent in those working than in the general population [24]. Nevertheless, the number of prevalent cases in MENA was higher in males, which highlights the need for future studies to explore the reasons (e.g. occupational exposures) for this finding. Consistent with the findings at the global level, the age-standardised prevalence rate of LBP in MENA peaked in the 80–84 age group, for both sexes, and then started to decrease [8]. In 2019, after the age of 40, the sex differences in the prevalent counts started to decrease in MENA and after 75 years of age, females had more prevalent cases than males. This difference could be due to the fact that disc space narrowing and the presence of osteophytes are significantly associated with chronic LBP [25]. Since disc degeneration is accelerated in postmenopausal women, they are at higher risk for the development of LBP, than premenopausal women [26].

Our report showed no overall association between SDI and the age-standardised YLD rate, over the period 1990–2019, although the age-standardised YLD was higher in countries with an SDI between 0.4 and 0.75. There was a similar association between the burden of LBP and SDI in most other GBD regions between 1990 and 2019. In contrast, at the global level there was an overall positive association between SDI and the burden of LBP [8].

The present study is the first to report the burden of LBP in the MENA region by age, sex, and SDI, using the most recent iteration of the GBD project. Nevertheless, our study has several limitations that should be taken into account when interpreting our findings. Firstly, the GBD uses modelled estimates instead of primary data. Furthermore, differences in the definition of LBP and the lack of an appropriate survey for gathering data on the burden and prevalence of LBP in many countries is another major limitation of the GBD study. For instance, an article by Maher et al. on LBP reported that only about 16% of countries had at least one report that used an appropriate measurement tool, so the prevalence data across countries and years remains sparse [27]. Furthermore, there are some unclear points in the results. For instance, Turkey had a very sudden and suspicious drop in the last 2 years of observation. This dramatic change may not be valid since there is no explanation for a change of over 10% in just 2 years when the region has only changed by 6% in 30 years. Secondly, the DWs that represent the severity of the disease, and were used to compute the YLD, were derived from the six LBP health states, which were based on the United States Health Service data [6]. Therefore, the LBP burden in MENA should be interpreted with some caution. Thirdly, we only assessed the patterns of LBP by sex and socioeconomic status, but there are other factors that are associated with LBP, which were not included in the present study, such as occupation, religion, education, social capital, race/ethnicity, culture and language [28]. Therefore, reporting the burden of LBP by religion, culture and race/ethnicity should be considered in future GBD iterations. Fourthly, the burden of LBP reported for each country might not be generalisable to all provinces of that country, as the subnational burden was not reported for any of the MENA countries.

## Conclusions

The burden of LBP has decreased in MENA over the last 30 years, although the decrease was lower than that found at the global level, especially among women. Although there were inter-country variations in the change in the burden of LBP over the measurement period, reductions were seen in all countries. This study highlights the fact that policy makers should initiate prevention programmes at early ages in order to reduce the incidence of LBP in the following decades of their lives. Furthermore, these interventions should prioritise females up to 20 years of age and males of all ages, especially in countries with high prevalence and incidence rates of LBP. Future research should focus on resolving the limitations of the present study, in particular the development of improved DWs based upon data from the MENA region.

## Supplementary Information

**Additional file 1:** **Table S1.** GATHER checklist.

**Additional file 2:** **Table S2.** Sequelae for low back pain and the associated disability weights from the Global Burden of Disease 2019 Study.

**Additional file 3:** **Table S3.** Prevalence of low back pain in 1990 and 2019 for both sexes and percentage change in age-standardised rates (ASRs) per 100,000 in the North Africa and the Middle East region (Generated from data available from http://ghdx.healthdata.org/gbd-results-tool).

**Additional file 4:** **Table S4.** Incidence of low back pain in 1990 and 2019 for both sexes and percentage change in age-standardised rates (ASRs) per 100,000 in the North Africa and the Middle East region (Generated from data available from http://ghdx.healthdata.org/gbd-results-tool).

**Additional file 5:** **Table S5.** YLDs due to low back pain in 1990 and 2019 for both sexes and percentage change in age-standardised rates (ASRs) per 100,000 in the North Africa and the Middle East region (Generated from data available from http://ghdx.healthdata.org/gbd-results-tool).

**Additional file 6:** **Fig. S1.** Age-standardised incidence rate of low back pain (per 100,000 population) in the Middle East and North Africa region in 2019, by sex and country. (Generated from data available from http://ghdx.healthdata.org/gbd-results-tool).

**Additional file 7:** **Fig. S2.** Age-standardised YLDs rate of low back pain (per 100,000 population) in the Middle East and North Africa region in 2019, by sex and country. YLD = years lived with disability. (Generated from data available from http://ghdx.healthdata.org/gbd-results-tool).

**Additional file 8:** **Fig. S3.** The percentage change in the age-standardised incidence of low back pain in the Middle East and North Africa region from 1990 to 2019, by sex and country. (Generated from data available from http://ghdx.healthdata.org/gbd-results-tool).

**Additional file 9:** **Fig. S4.** The percentage change in the age-standardised YLDs of low back pain in the Middle East and North Africa region from 1990 to 2019, by sex and country. YLD = years lived with disability. (Generated from data available from http://ghdx.healthdata.org/gbd-results-tool).

**Additional file 10:** **Fig. S5.** Numbers of incident cases and incidence rate of low back pain per 100,000 population in the Middle East and North Africa region, by age and sex in 2019; Dotted and dashed lines indicate 95% upper and lower uncertainty intervals for the incidence rates per 100,000 population, respectively. The solid lines represent the point estimation for the incidence rate per 100,000 population. (Generated from data available from http://ghdx.healthdata.org/gbd-results-tool).

**Additional file 11:** **Fig. S6.** Numbers of YLDs and YLD rate of low back pain per 100,000 population in the Middle East and North Africa region, by age and sex in 2019; Dotted and dashed lines indicate 95% upper and lower uncertainty intervals for the YLD rates per 100,000 population, respectively. The solid lines represent the point estimation for the YLD rate per 100,000 population. YLD = years lived with disability. (Generated from data available from http://ghdx.healthdata.org/gbd-results-tool).

## Data Availability

The datasets generated and/or analysed during the current study are available in the Global Burden of Disease (GBD) database repository, http://ghdx.healthdata.org/gbd-results-tool.
