# Supplementary material for: The burden of low back pain and its association with socio-demographic variables in the Middle East and North Africa region, 1990–2019
Source: BMC Musculoskelet Disord. 2023 Jan 23;24:59. doi: 10.1186/s12891-023-06178-3 (PMC9869505; doi:10.1186/s12891-023-06178-3)
Supplement: Supplementary file 2 — Additional file 2: Table S2. Sequelae for low back pain and the associated disability weights from the Global Burden of Disease 2019 Study. [file 12891_2023_6178_MOESM2_ESM.docx]

| **Table S2: Sequelae for low back pain and the associated disability weights from the Global Burden of Disease 2019 Study** | | |
| --- | --- | --- |
| **Severity level** | **Lay description** | **Disability weight**  **(95% CI)** |
| Mild | This person has mild back pain, which causes some difficulty dressing, standing, and lifting things. | 0.020  (0.011–0.035) |
| Moderate | This person has moderate back pain, which causes difficulty dressing, sitting, standing, walking, and lifting things. | 0.054  (0.035–0.079) |
| Severe without leg pain | This person has severe back pain, which causes difficulty dressing, sitting, standing, walking, and lifting things. The person sleeps poorly and feels worried. | 0.272  (0.182–0.373) |
| Severe with leg pain | This person has severe back and leg pain, which causes difficulty dressing, sitting, standing, walking, and lifting things. The person sleeps poorly and feels worried. | 0.325  (0.219–0.446) |
| Most severe without leg pain | This person has constant back pain, which causes difficulty dressing, sitting, standing, walking, and lifting things. The person sleeps poorly, is worried, and has lost some enjoyment in life. | 0.372  (0.250–0.506) |
| Most severe with leg pain | This person has constant back and leg pain, which causes difficulty dressing, sitting, standing, walking, and lifting things. The person sleeps poorly, is worried, and has lost some enjoyment in life. | 0.384  (0.256–0.518) |
