# Supplementary material for: The burden of low back pain and its association with socio-demographic variables in the Middle East and North Africa region, 1990–2019
Source: BMC Musculoskelet Disord. 2023 Jan 23;24:59. doi: 10.1186/s12891-023-06178-3 (PMC9869505; doi:10.1186/s12891-023-06178-3)
Supplement: Supplementary file 3 — Additional file 3: Table S3. Prevalence of low back pain in 1990 and 2019 for both sexes and percentage change in age-standardised rates (ASRs) per 100,000 in the North Africa and the Middle East region (Generated from data available from http://ghdx.healthdata.org/gbd-results-tool). [file 12891_2023_6178_MOESM3_ESM.docx]

| **Table S3: Prevalence of low back pain in 1990 and 2019 for both sexes and percentage change in age-standardised rates (ASRs) per 100,000 in the North Africa and the Middle East region**  **(Generated from data available from http://ghdx.healthdata.org/gbd-results-tool)** | | | | | |
| --- | --- | --- | --- | --- | --- |
|  | **1990** | | **2019** | | **Percentage change in ASRs per 100,000** |
|  | **No (95% UI)** | **ASRs per 100,000 (95% UI)** | **No (95% UI)** | **ASRs per 100,000 (95% UI)** |  |
| **North Africa and Middle East** | **20077912 (17626717 , 22491145)** | **8140.7 (7212.6 , 9121.2)** | **43239039 (37773370 , 48819120)** | **7668.2 (6798 , 8636.3)** | **-5.8 (-7.4 , -4.3)** |
| **Afghanistan** | **594847 (520570 , 672492)** | **7239.8 (6332.1 , 8183.8)** | **1757626 (1520204 , 2005763)** | **7307 (6427 , 8281.8)** | **0.9 (-2.8 , 4.9)** |
| **Algeria** | **1298441 (1131655 , 1469536)** | **7380.1 (6483.1 , 8340.9)** | **2938809 (2560184 , 3336911)** | **7237.5 (6353.5 , 8175.9)** | **-1.9 (-5.8 , 1.9)** |
| **Bahrain** | **29377 (25045 , 33819)** | **7284.8 (6382.3 , 8249.3)** | **120196 (102199 , 139069)** | **7213.6 (6350.1 , 8149)** | **-1 (-4.7 , 3.1)** |
| **Egypt** | **3024108 (2632482 , 3419903)** | **7304.1 (6429.9 , 8283)** | **6364141 (5533169 , 7233281)** | **7461.2 (6540.7 , 8414.6)** | **2.2 (-1.6 , 6.5)** |
| **Iran (Islamic Republic of)** | **3701159 (3239976 , 4181658)** | **9377 (8310.7 , 10579.1)** | **7415044 (6524554 , 8393689)** | **8486.5 (7533.8 , 9528.3)** | **-9.5 (-10.6 , -8.3)** |
| **Iraq** | **849931 (738667 , 963878)** | **7400.5 (6516.6 , 8371.4)** | **2476373 (2157322 , 2802292)** | **7172.3 (6322.7 , 8116.5)** | **-3.1 (-6.5 , 0.6)** |
| **Jordan** | **175566 (151927 , 199969)** | **7338.1 (6460.5 , 8313.2)** | **717146 (622952 , 816397)** | **7253.9 (6391.6 , 8222.1)** | **-1.1 (-4.7 , 2.5)** |
| **Kuwait** | **103308 (87978 , 118760)** | **7389.3 (6477.8 , 8369.5)** | **346361 (297418 , 398409)** | **7412 (6519.9 , 8339.5)** | **0.3 (-3.5 , 4.4)** |
| **Lebanon** | **173494 (151466 , 197171)** | **6543.6 (5745.4 , 7421.4)** | **346477 (304628 , 392389)** | **6493.5 (5721.5 , 7349.7)** | **-0.8 (-4.2 , 2.8)** |
| **Libya** | **205433 (179161 , 234665)** | **7280 (6408 , 8232.9)** | **490208 (423628 , 561130)** | **7103.7 (6233.9 , 8024.1)** | **-2.4 (-6.2 , 1.3)** |
| **Morocco** | **1438469 (1268964 , 1616583)** | **7535.7 (6690.3 , 8437.8)** | **2770031 (2428118 , 3142593)** | **7717.5 (6818.5 , 8677.7)** | **2.4 (-1.6 , 6.6)** |
| **Oman** | **96620 (83208 , 110459)** | **7276.5 (6394 , 8232.6)** | **307584 (259823 , 357611)** | **7209.6 (6333.4 , 8186.6)** | **-0.9 (-4.7 , 3.4)** |
| **Palestine** | **93688 (81682 , 107049)** | **7355.2 (6457.3 , 8379.5)** | **265697 (232238 , 301139)** | **7102.3 (6257.6 , 7999.9)** | **-3.4 (-7.1 , 0.3)** |
| **Qatar** | **28180 (23482 , 33028)** | **7477 (6538 , 8484.4)** | **222755 (188144 , 258441)** | **7483.1 (6566.5 , 8458.3)** | **0.1 (-3.8 , 4.7)** |
| **Saudi Arabia** | **782994 (674611 , 893405)** | **7146.8 (6264.7 , 8096.4)** | **2553569 (2178915 , 2947793)** | **7170.1 (6283.2 , 8113.1)** | **0.3 (-3.3 , 4.1)** |
| **Sudan** | **954982 (830092 , 1084953)** | **7095.3 (6218.3 , 7996.7)** | **2093653 (1817650 , 2382925)** | **7006.6 (6190.2 , 7926.1)** | **-1.3 (-4.7 , 2.3)** |
| **Syrian Arab Republic** | **605317 (528061 , 686618)** | **7474.2 (6598.7 , 8480.5)** | **1007021 (871605 , 1147942)** | **7160.2 (6255 , 8108.7)** | **-4.2 (-8 , -0.2)** |
| **Tunisia** | **456037 (401454 , 514654)** | **6991.4 (6173.7 , 7891.1)** | **897946 (783942 , 1024027)** | **7022.1 (6160.7 , 7987.7)** | **0.4 (-3.4 , 4.6)** |
| **Turkey** | **4741284 (4167295 , 5294482)** | **9853.2 (8758.4 , 10990.9)** | **7730835 (6788734 , 8691678)** | **8453.5 (7457.1 , 9478.7)** | **-14.2 (-19.5 , -9.2)** |
| **United Arab Emirates** | **105906 (89480 , 123414)** | **7134 (6290.1 , 8022.1)** | **770334 (641479 , 919311)** | **7101.6 (6223.6 , 8047.2)** | **-0.5 (-4.9 , 4.4)** |
| **Yemen** | **605267 (525094 , 688300)** | **7621.6 (6668.3 , 8630.5)** | **1603302 (1388518 , 1831446)** | **7208.7 (6336 , 8120.3)** | **-5.4 (-9.1 , -1.8)** |
