# Supplementary material for: The burden of low back pain and its association with socio-demographic variables in the Middle East and North Africa region, 1990–2019
Source: BMC Musculoskelet Disord. 2023 Jan 23;24:59. doi: 10.1186/s12891-023-06178-3 (PMC9869505; doi:10.1186/s12891-023-06178-3)
Supplement: Supplementary file 4 — Additional file 4: Table S4. Incidence of low back pain in 1990 and 2019 for both sexes and percentage change in age-standardised rates (ASRs) per 100,000 in the North Africa and the Middle East region (Generated from data available from http://ghdx.healthdata.org/gbd-results-tool). [file 12891_2023_6178_MOESM4_ESM.docx]

| **Table S4: Incidence of low back pain in 1990 and 2019 for both sexes and percentage change in age-standardised rates (ASRs) per 100,000 in the North Africa and the Middle East region**  **(Generated from data available from http://ghdx.healthdata.org/gbd-results-tool)** | | | | | |
| --- | --- | --- | --- | --- | --- |
|  | **1990** | | **2019** | | **Percentage change in ASRs per 100,000** |
|  | **No (95% UI)** | **ASRs per 100,000 (95% UI)** | **No (95% UI)** | **ASRs per 100,000 (95% UI)** |  |
| **North Africa and Middle East** | **8572369 (7559451 , 9724393)** | **3364.2 (2983.1 , 3790.4)** | **18336870 (16080397 , 20926523)** | **3215.9 (2838.8 , 3638.3)** | **-4.4 (-5.5 , -3.4)** |
| **Afghanistan** | **260642 (227505 , 297810)** | **3090.4 (2704.5 , 3513.8)** | **781631 (677351 , 895750)** | **3108.2 (2743.7 , 3521.5)** | **0.6 (-2.4 , 3.5)** |
| **Algeria** | **572095 (498495 , 654391)** | **3139.3 (2769.1 , 3562.8)** | **1257427 (1097529 , 1445308)** | **3081.2 (2713.3 , 3498)** | **-1.8 (-4.7 , 1.1)** |
| **Bahrain** | **12878 (11007 , 15128)** | **3113.8 (2739.5 , 3517.3)** | **51265 (43818 , 59721)** | **3082.6 (2707.7 , 3504)** | **-1 (-4.3 , 2.1)** |
| **Egypt** | **1324330 (1163296 , 1509285)** | **3114.8 (2755.6 , 3527.4)** | **2738368 (2402636 , 3135984)** | **3155.7 (2789.9 , 3568.5)** | **1.3 (-1.6 , 4.3)** |
| **Iran (Islamic Republic of)** | **1564670 (1374347 , 1779555)** | **3811.3 (3375.5 , 4288)** | **3050709 (2686650 , 3487116)** | **3493 (3092.5 , 3949.5)** | **-8.4 (-9.2 , -7.5)** |
| **Iraq** | **374518 (326526 , 427240)** | **3141.4 (2767.8 , 3566)** | **1079909 (949882 , 1240336)** | **3058.3 (2709.3 , 3476.3)** | **-2.6 (-5.4 , 0)** |
| **Jordan** | **78275 (68084 , 89952)** | **3127.5 (2760.4 , 3556.7)** | **312024 (271793 , 358356)** | **3094.6 (2725.9 , 3511.3)** | **-1.1 (-4.1 , 1.9)** |
| **Kuwait** | **45150 (38775 , 52693)** | **3147.7 (2771.6 , 3558.7)** | **147167 (126465 , 171814)** | **3140.3 (2778.6 , 3552.8)** | **-0.2 (-3.3 , 2.5)** |
| **Lebanon** | **77070 (67327 , 88128)** | **2860.3 (2502.6 , 3271.5)** | **150708 (132419 , 171751)** | **2830.3 (2484.6 , 3214.3)** | **-1 (-4 , 1.8)** |
| **Libya** | **91057 (79235 , 103820)** | **3109.1 (2745.7 , 3534.1)** | **210985 (183586 , 243608)** | **3042 (2684.6 , 3459)** | **-2.2 (-5 , 1.1)** |
| **Morocco** | **625768 (551155 , 712360)** | **3189.3 (2831.4 , 3592.4)** | **1165371 (1027188 , 1327666)** | **3230.5 (2860.1 , 3658.1)** | **1.3 (-1.5 , 4.3)** |
| **Oman** | **42911 (37063 , 49850)** | **3116.5 (2744.2 , 3537.7)** | **133636 (113171 , 158197)** | **3087.2 (2716.5 , 3502.3)** | **-0.9 (-4 , 2.4)** |
| **Palestine** | **41514 (36219 , 47407)** | **3127.2 (2761.5 , 3556.7)** | **117259 (102576 , 134388)** | **3042.9 (2687.5 , 3462.5)** | **-2.7 (-5.6 , 0.7)** |
| **Qatar** | **12323 (10454 , 14658)** | **3195.8 (2831.2 , 3625.5)** | **95630 (81013 , 114716)** | **3184.1 (2795.4 , 3622.6)** | **-0.4 (-3.7 , 2.9)** |
| **Saudi Arabia** | **349155 (303372 , 403660)** | **3077.5 (2712.1 , 3496.1)** | **1104496 (943692 , 1291967)** | **3071.7 (2708.8 , 3479.2)** | **-0.2 (-3.3 , 2.9)** |
| **Sudan** | **423957 (369300 , 485243)** | **3047.1 (2685.5 , 3468.2)** | **927368 (803298 , 1067815)** | **3010.1 (2654 , 3429.2)** | **-1.2 (-4 , 1.4)** |
| **Syrian Arab Republic** | **268079 (232126 , 306369)** | **3170 (2800.1 , 3587.7)** | **433623 (378477 , 495509)** | **3056.3 (2686.2 , 3463.2)** | **-3.6 (-6.3 , -0.8)** |
| **Tunisia** | **201354 (176620 , 227349)** | **3012.9 (2658.3 , 3389.8)** | **382940 (337333 , 438013)** | **3009.4 (2660.6 , 3420.7)** | **-0.1 (-3.2 , 3.5)** |
| **Turkey** | **1886092 (1668214 , 2119448)** | **3816.3 (3409 , 4263.9)** | **3140050 (2767927 , 3566643)** | **3452.6 (3052.3 , 3899.1)** | **-9.5 (-13.4 , -5.8)** |
| **United Arab Emirates** | **46850 (39438 , 55496)** | **3078.4 (2715.5 , 3478.8)** | **330457 (271005 , 401767)** | **3053 (2672 , 3475.2)** | **-0.8 (-4.2 , 2.9)** |
| **Yemen** | **267916 (233314 , 307656)** | **3214.4 (2823.5 , 3647.4)** | **707218 (614181 , 811161)** | **3072.8 (2702.7 , 3471.5)** | **-4.4 (-7.3 , -1.2)** |
