# Supplementary material for: The burden of low back pain and its association with socio-demographic variables in the Middle East and North Africa region, 1990–2019
Source: BMC Musculoskelet Disord. 2023 Jan 23;24:59. doi: 10.1186/s12891-023-06178-3 (PMC9869505; doi:10.1186/s12891-023-06178-3)
Supplement: Supplementary file 5 — Additional file 5: Table S5. YLDs due to low back pain in 1990 and 2019 for both sexes and percentage change in age-standardised rates (ASRs) per 100,000 in the North Africa and the Middle East region (Generated from data available from http://ghdx.healthdata.org/gbd-results-tool). [file 12891_2023_6178_MOESM5_ESM.docx]

| **Table S5: YLDs due to low back pain in 1990 and 2019 for both sexes and percentage change in age-standardised rates (ASRs) per 100,000 in the North Africa and the Middle East region**  **(Generated from data available from http://ghdx.healthdata.org/gbd-results-tool)** | | | | | |
| --- | --- | --- | --- | --- | --- |
|  | **1990** | | **2019** | | **Percentage change in ASRs per 100,000** |
|  | **No (95% UI)** | **ASRs per 100,000 (95% UI)** | **No (95% UI)** | **ASRs per 100,000 (95% UI)** |  |
| **North Africa and Middle East** | **2274265 (1590072 , 3033870)** | **916.6 (648.6 , 1222.1)** | **4899300 (3404485 , 6533347)** | **862 (605.5 , 1153.3)** | **-6 (-7.7 , -4.2)** |
| **Afghanistan** | **65527 (46448 , 88105)** | **795.8 (561.2 , 1062.5)** | **196023 (136064 , 264247)** | **803.6 (568 , 1075.4)** | **1 (-3.6 , 5.7)** |
| **Algeria** | **147530 (103226 , 198760)** | **834.2 (590 , 1121.1)** | **333813 (230155 , 447252)** | **816.5 (567.8 , 1094)** | **-2.1 (-6.3 , 2.3)** |
| **Bahrain** | **3363 (2297 , 4545)** | **818.9 (572.7 , 1102.2)** | **13742 (9477 , 18769)** | **808.9 (568.9 , 1083.6)** | **-1.2 (-5.5 , 3.2)** |
| **Egypt** | **343510 (237297 , 460011)** | **824.8 (575.2 , 1093.7)** | **724668 (504744 , 974424)** | **842.9 (590.7 , 1130.1)** | **2.2 (-2.2 , 6.9)** |
| **Iran (Islamic Republic of)** | **418229 (292449 , 558428)** | **1051.4 (738.5 , 1404.5)** | **837779 (583888 , 1128834)** | **951.9 (668.9 , 1275.2)** | **-9.5 (-10.7 , -8.2)** |
| **Iraq** | **95284 (67114 , 127949)** | **824.1 (577.4 , 1099.8)** | **279601 (193899 , 377243)** | **802.3 (560.9 , 1078.3)** | **-2.6 (-6.6 , 1.3)** |
| **Jordan** | **19963 (13806 , 26946)** | **828.2 (581.6 , 1110)** | **81779 (56682 , 108985)** | **819.8 (570.5 , 1091.2)** | **-1 (-4.9 , 3.2)** |
| **Kuwait** | **11860 (8090 , 16137)** | **836.4 (585.9 , 1121)** | **39639 (26978 , 53817)** | **835.7 (589.3 , 1121)** | **-0.1 (-4.3 , 4.3)** |
| **Lebanon** | **19555 (13876 , 26494)** | **734.3 (518.3 , 997)** | **38867 (27267 , 52121)** | **727.2 (511.5 , 966)** | **-1 (-4.7 , 3.1)** |
| **Libya** | **23310 (16407 , 31455)** | **821.8 (575.2 , 1113.4)** | **55476 (38326 , 75270)** | **796.3 (558.9 , 1069.2)** | **-3.1 (-7.1 , 1.1)** |
| **Morocco** | **163005 (113687 , 217475)** | **849.5 (595.9 , 1139.8)** | **313322 (220856 , 423475)** | **868 (610.1 , 1167)** | **2.2 (-2.2 , 6.9)** |
| **Oman** | **11049 (7611 , 14907)** | **820.1 (576 , 1098.4)** | **35426 (24025 , 48333)** | **812.7 (568.2 , 1092.1)** | **-0.9 (-5.3 , 3.9)** |
| **Palestine** | **10529 (7325 , 14173)** | **823.5 (573.5 , 1101.8)** | **29959 (20907 , 39885)** | **793.3 (560.7 , 1063.7)** | **-3.7 (-7.6 , 0.6)** |
| **Qatar** | **3248 (2182 , 4429)** | **841.5 (587.9 , 1119.7)** | **25695 (17541 , 35014)** | **839.5 (592.4 , 1132.8)** | **-0.2 (-4.8 , 5.1)** |
| **Saudi Arabia** | **89128 (61307 , 119808)** | **802.6 (559.7 , 1069.9)** | **291699 (199992 , 397000)** | **803 (568.3 , 1071.7)** | **0 (-4 , 4.4)** |
| **Sudan** | **108047 (75327 , 145894)** | **799.1 (563.1 , 1076.5)** | **237414 (164627 , 316341)** | **788.3 (553.5 , 1045.1)** | **-1.3 (-5.2 , 2.6)** |
| **Syrian Arab Republic** | **68670 (48219 , 91395)** | **844.2 (591.1 , 1118.9)** | **113375 (79653 , 152271)** | **802.3 (559.6 , 1070.6)** | **-5 (-9.1 , -0.7)** |
| **Tunisia** | **51847 (36397 , 68764)** | **791 (554.1 , 1058.8)** | **101525 (71422 , 135332)** | **791.5 (553.5 , 1052.4)** | **0.1 (-4.2 , 5)** |
| **Turkey** | **538716 (378475 , 723182)** | **1114.4 (784.7 , 1489.2)** | **874588 (608052 , 1178266)** | **953.6 (671.3 , 1283.5)** | **-14.4 (-19.9 , -8.9)** |
| **United Arab Emirates** | **12221 (8329 , 16404)** | **804.5 (566.9 , 1070.6)** | **89026 (60322 , 122762)** | **798.9 (559.5 , 1067.8)** | **-0.7 (-5.5 , 4.4)** |
| **Yemen** | **68145 (47566 , 91054)** | **852.9 (601.7 , 1143)** | **180905 (124718 , 242346)** | **807.2 (563.7 , 1075.2)** | **-5.4 (-9.3 , -1.4)** |
