# Supplementary material for: The burden of low back pain and its association with socio-demographic variables in the Middle East and North Africa region, 1990–2019
Source: BMC Musculoskelet Disord. 2023 Jan 23;24:59. doi: 10.1186/s12891-023-06178-3 (PMC9869505; doi:10.1186/s12891-023-06178-3)
Supplement: Supplementary file 6 — Additional file 6: Fig. S1. Age-standardised incidence rate of low back pain (per 100,000 population) in the Middle East and North Africa region in 2019, by sex and country. (Generated from data available from http://ghdx.healthdata.org/gbd-results-tool). [file 12891_2023_6178_MOESM6_ESM.pdf]

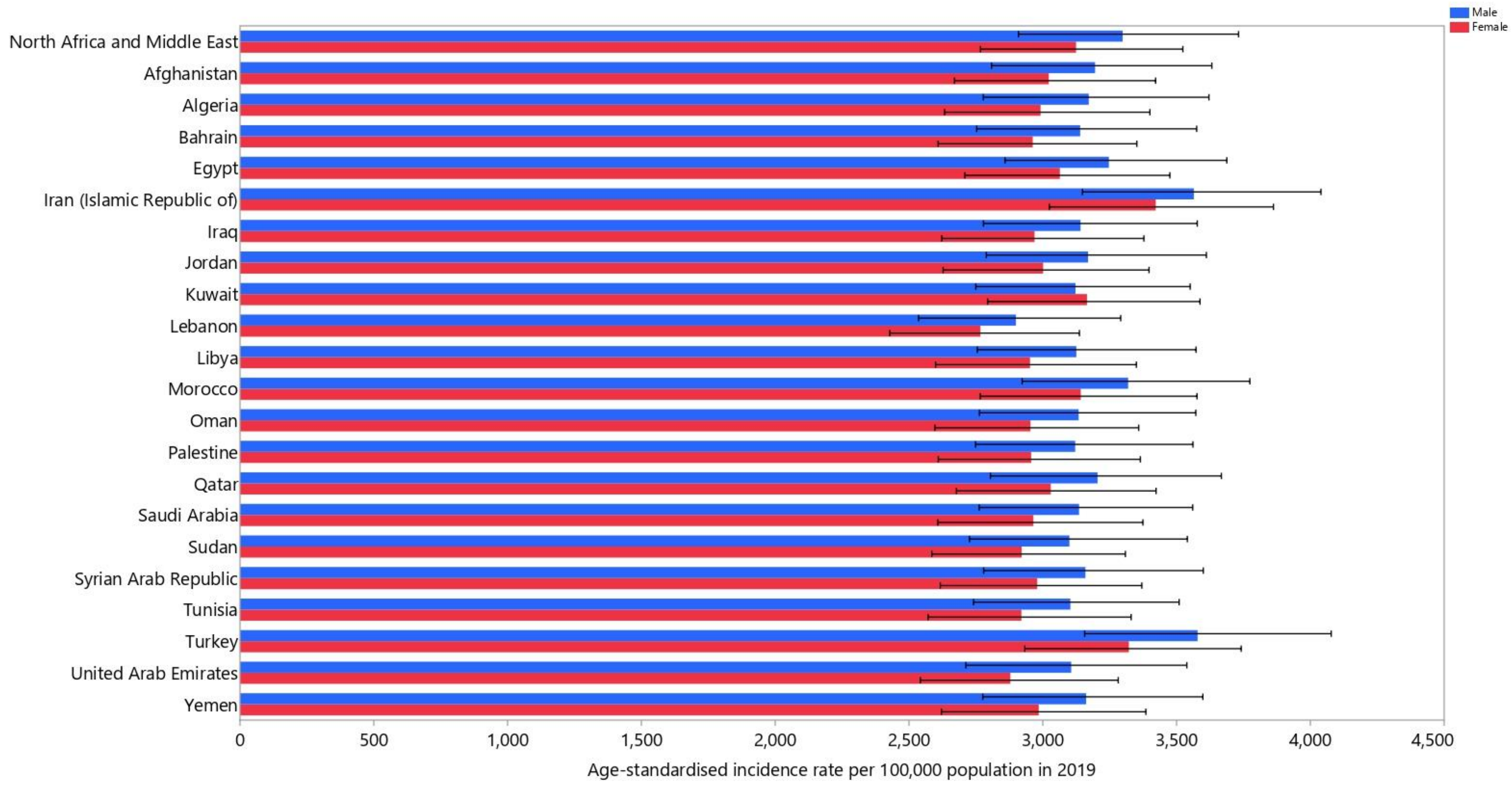

Figure S1: Age-standardised incidence rate of low back pain (per 100,000 population) in the Middle East and North Africa region in 2019, by sex and country. (Generated from data available from http://ghdx.healthdata.org/gbd-results-tool).
