# Supplementary material for: The burden of low back pain and its association with socio-demographic variables in the Middle East and North Africa region, 1990–2019
Source: BMC Musculoskelet Disord. 2023 Jan 23;24:59. doi: 10.1186/s12891-023-06178-3 (PMC9869505; doi:10.1186/s12891-023-06178-3)
Supplement: Supplementary file 9 — Additional file 9: Fig. S4. The percentage change in the age-standardised YLDs of low back pain in the Middle East and North Africa region from 1990 to 2019, by sex and country. YLD = years lived with disability. (Generated from data available from http://ghdx.healthdata.org/gbd-results-tool). [file 12891_2023_6178_MOESM9_ESM.pdf]

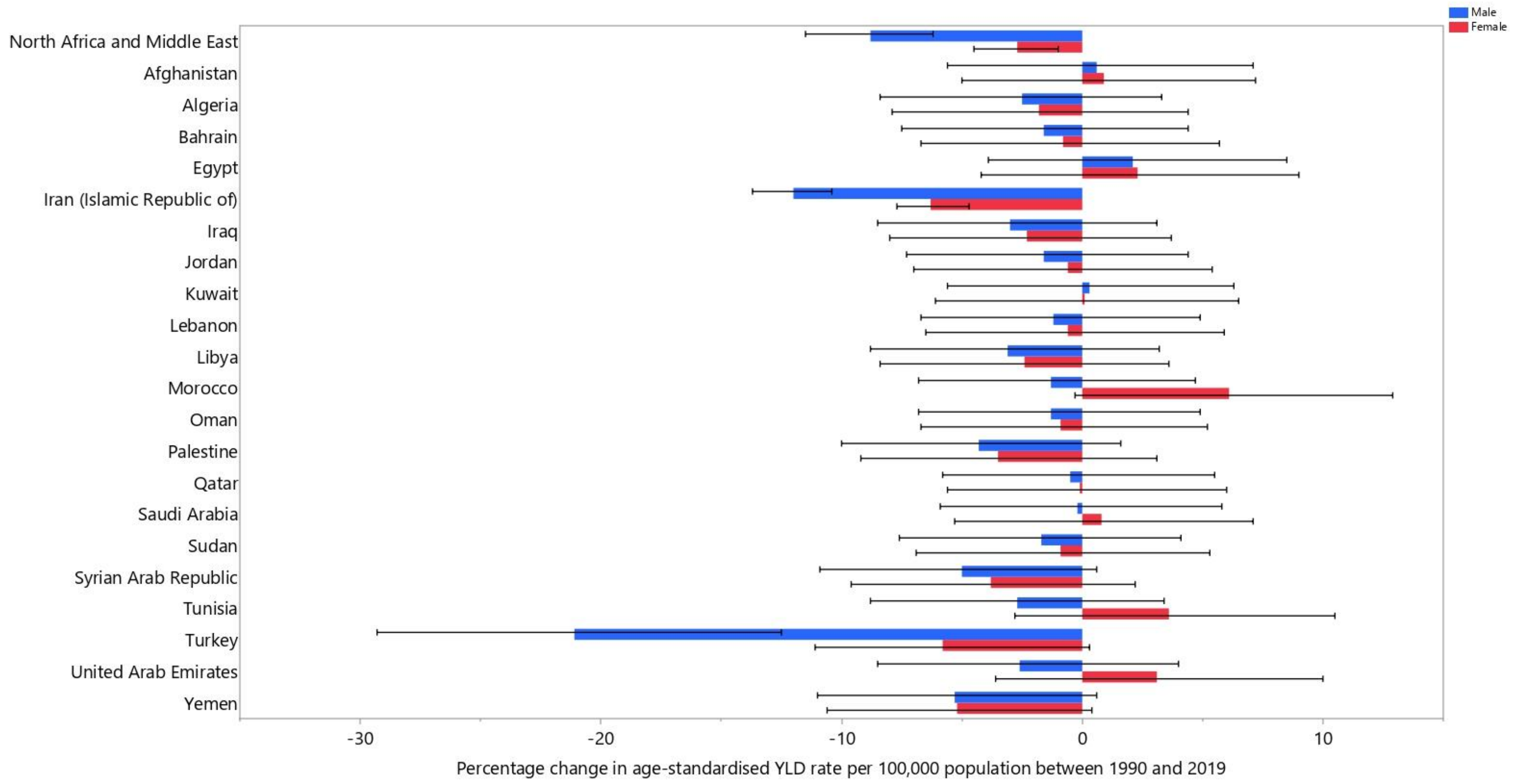

Figure S4: The percentage change in the age-standardised YLDs of low back pain in the Middle East and North Africa region from 1990 to 2019, by sex and country. YLD= years lived with disability. (Generated from data available from http://ghdx.healthdata.org/gbd-results-tool).
