# Supplementary material for: The burden of low back pain and its association with socio-demographic variables in the Middle East and North Africa region, 1990–2019
Source: BMC Musculoskelet Disord. 2023 Jan 23;24:59. doi: 10.1186/s12891-023-06178-3 (PMC9869505; doi:10.1186/s12891-023-06178-3)
Supplement: Supplementary file 10 — Additional file 10: Fig. S5. Numbers of incident cases and incidence rate of low back pain per 100,000 population in the Middle East and North Africa region, by age and sex in 2019; Dotted and dashed lines indicate 95% upper and lower uncertainty intervals for the incidence rates per 100,000 population, respectively. The solid lines represent the point estimation for the incidence rate per 100,000 population. (Generated from data available from http://ghdx.healthdata.org/gbd-results-tool). [file 12891_2023_6178_MOESM10_ESM.pdf]

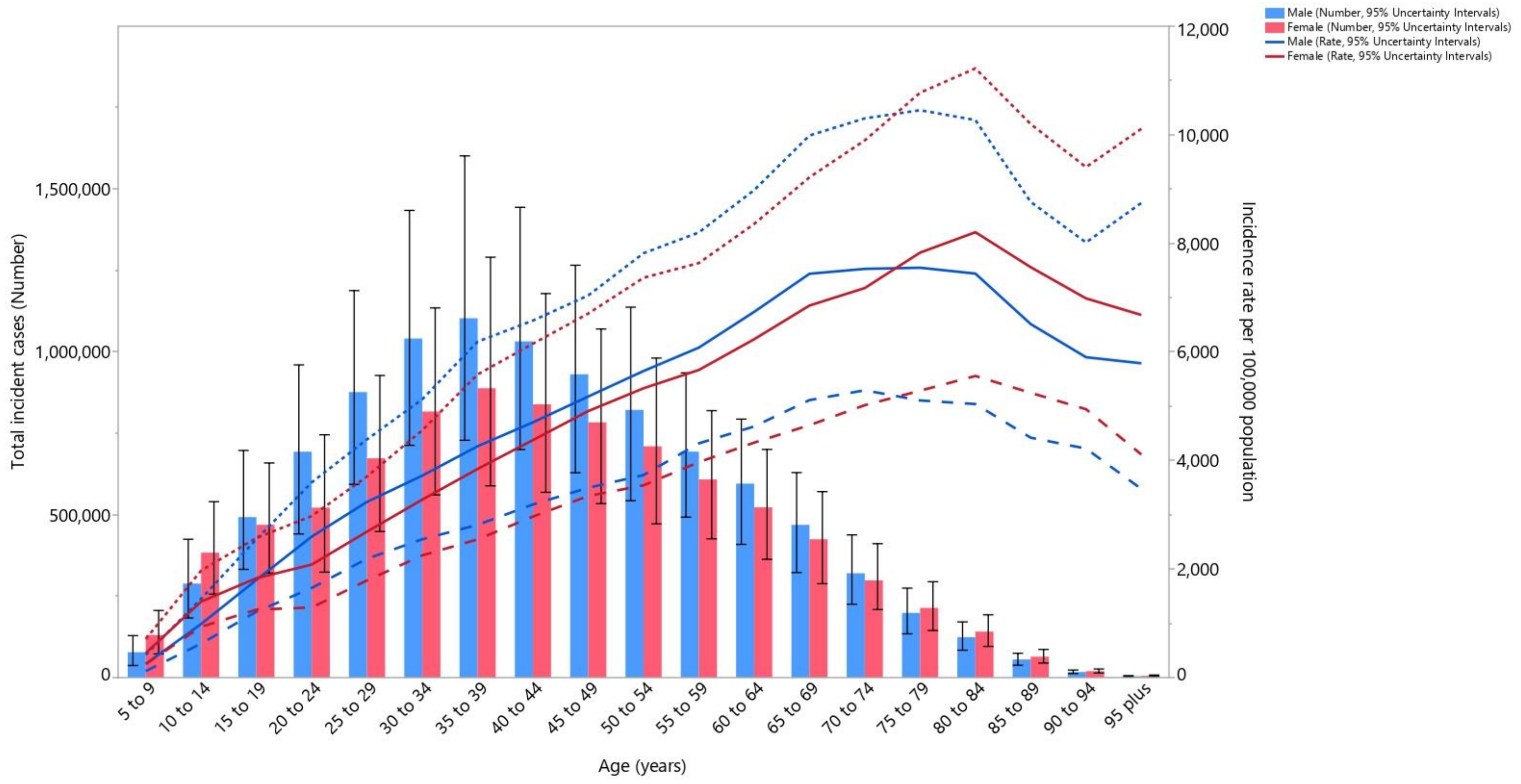

Figure S5: Numbers of incident cases and incidence rate of low back pain per 100,000 population in the Middle East and North Africa region, by age and sex in 2019; Dotted and dashed lines indicate 95% upper and lower uncertainty intervals for the incidence rates per 100,000 population, respectively. The solid lines represent the point estimation for the incidence rate per 100,000 population. (Generated from data available from http://ghdx.healthdata.org/gbd-results-tool).
