# Supplementary material for: The burden of low back pain and its association with socio-demographic variables in the Middle East and North Africa region, 1990–2019
Source: BMC Musculoskelet Disord. 2023 Jan 23;24:59. doi: 10.1186/s12891-023-06178-3 (PMC9869505; doi:10.1186/s12891-023-06178-3)
Supplement: Supplementary file 11 — Additional file 11: Fig. S6. Numbers of YLDs and YLD rate of low back pain per 100,000 population in the Middle East and North Africa region, by age and sex in 2019; Dotted and dashed lines indicate 95% upper and lower uncertainty intervals for the YLD rates per 100,000 population, respectively. The solid lines represent the point estimation for the YLD rate per 100,000 population. YLD = years lived with disability. (Generated from data available from http://ghdx.healthdata.org/gbd-results-tool). [file 12891_2023_6178_MOESM11_ESM.pdf]

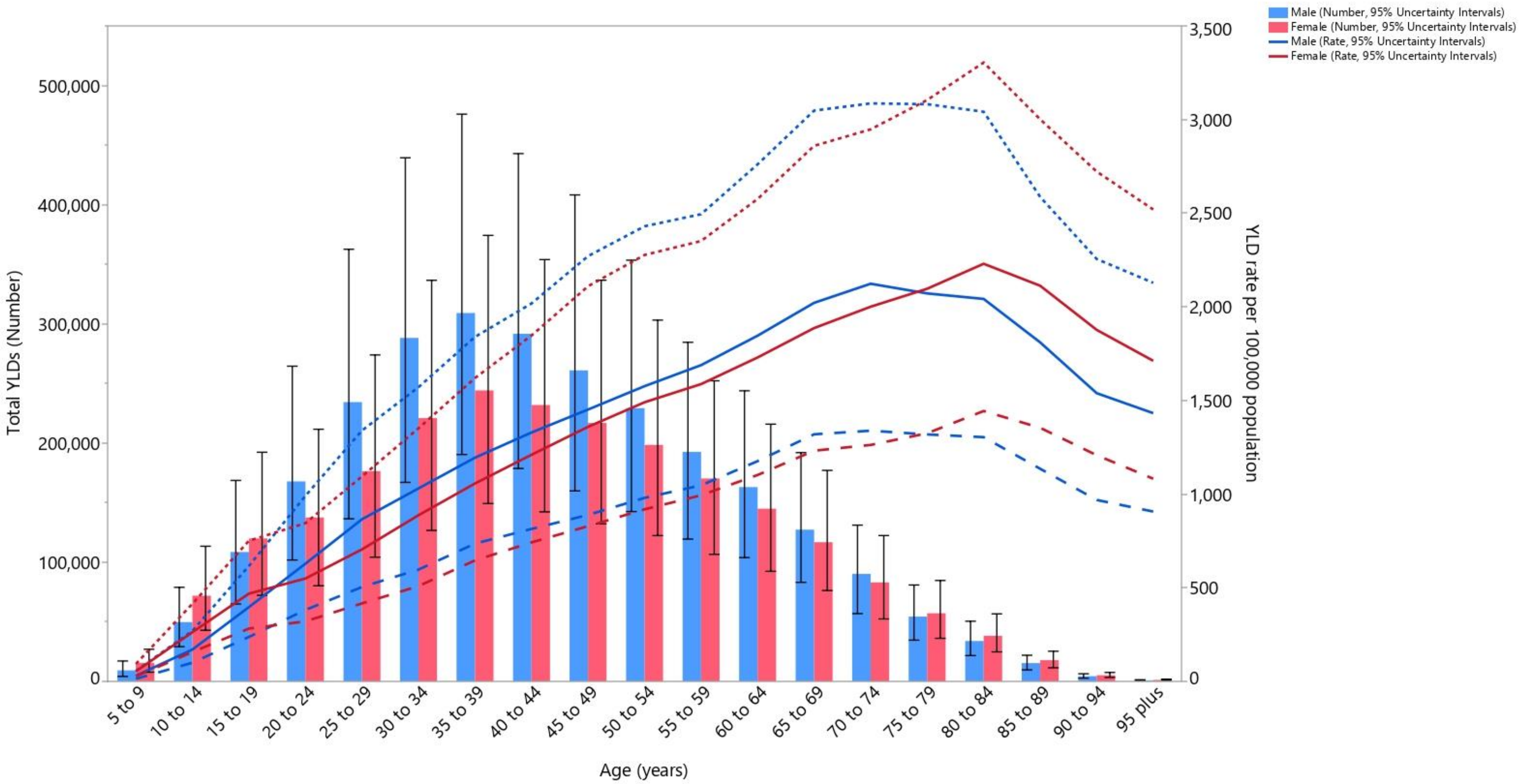

Figure S6: Numbers of YLDs and YLD rate of low back pain per 100,000 population in the Middle East and North Africa region, by age and sex in 2019; Dotted and dashed lines indicate 95% upper and lower uncertainty intervals for the YLD rates per 100,000 population, respectively. The solid lines represent the point estimation for the YLD rate per 100,000 population. YLD= years lived with disability. (Generated from data available from http://ghdx.healthdata.org/gbd-results-tool).
